# Interdigital Hyperplasia in Holstein Cattle Is Associated With a Missense Mutation in the Signal Peptide Region of the Tyrosine-Protein Kinase Transmembrane Receptor Gene

**DOI:** 10.3389/fgene.2019.01157

**Published:** 2019-11-13

**Authors:** Xuying Zhang, Hermann H. Swalve, René Pijl, Frank Rosner, Monika Wensch-Dorendorf, Bertram Brenig

**Affiliations:** ^1^Institute of Veterinary Medicine, University of Göttingen, Göttingen, Germany; ^2^Animal Breeding, Institute of Agricultural and Nutritional Sciences, Martin-Luther-University Halle-Wittenberg, Halle/Saale, Germany; ^3^Independent Researcher, Jever, Germany

**Keywords:** Claw disease, cattle, interdigital hyperplasia, limax, tyloma, receptor tyrosine kinase-like orphan receptor 2

## Abstract

Bovine interdigital hyperplasia (IH) is a typical disease of the foot with varying prevalence depending on age, breed, and environmental factors resulting in different degrees of lameness. In studies based on assessments of claw health status at time of hoof trimming and applying genetic-statistical models to analyze this data, IH consistently exhibits high estimates of heritability in the range of 0.30–0.40. Although some studies have identified chromosomal regions that could possibly harbor causative genes, a clear identification of molecular causes for IH is lacking. While analyzing the large database of claw health status as documented at time of hoof trimming, we identified one herd with extreme prevalence of IH of > 50% of affected Holstein dairy cows. This herd subsequently was chosen as the object of a detailed study. A total of n = 91 cows was assessed and revealed a prevalence of 59.3% and 38.5% for IH cases, documented as “one-sided” or “two-sided”, respectively. Cows were genotyped using the BovineSNP50 BeadChip. A genome wide association study revealed two significantly associated chromosomal positions (-log_10_P = 5.57) on bovine chromosome 8 (BTA8) located in intron 5 and downstream of the receptor tyrosine kinase-like orphan receptor 2 (*ROR2*) gene. As ROR2 plays a key role in ossification of the distal limbs and is associated with brachydactylies in humans, it was a reasonable candidate for IH. A comparative sequencing of the *ROR2* gene between cases and controls revealed two missense variants in exon 1 (NC_037335.1:g.85,905,534T > A, ARS-UCD1.2) and exon 9 (NC_037335.1:g.86,140,379A > G, ARS-UCD1.2), respectively. Genotyping of both variants in the cohort of 91 cattle showed that the exon 1 variant (rs377953295) remained significantly associated with IH (p < 0.0001) as a risk factor of the disease. This variant resulted in an amino acid exchange (ENSBTAP00000053765.2:p.Trp9Arg) in the N-terminal region of the ROR2 signal peptide which is necessary for proper topology of the polypeptide during translocation. Quantification of *ROR2* mRNA and ROR2 protein showed that the variant resulted in a significant suppression of *ROR2* expression in homozygous affected compared to wild type and carrier cows.

## Introduction

Foot diseases are a major and increasing health problem in dairy cattle and show rather high prevalence (Gernand et al., 2012). Often over 70% cows in dairy herds experience one or more front or hind claw lesions or deformities during their life (Smits et al., 1992; van der Waaij et al., 2005; Fjeldaas et al., 2006; van der Linde et al., 2010). Due to milk production losses and veterinary costs as well as culling of severe cases, feet and leg are of economical importance (Bruijnis et al., 2010; Schopke et al., 2013). Besides disorders like double sole, sole ulcer, sole hemorrhage, white line separation, and digital dermatitis, interdigital hyperplasia (IH) plays an important role with a prevalence of around 5%–14.2% (Smits et al., 1992; Somers et al., 2003; van der Waaij et al., 2005; van der Linde et al., 2010). Normally IH begins with a small and painless protrusion of the interdigital skin which sometimes can already be diagnosed at calf age. In the final stage clinical signs of IH are firm tumor like masses found in the interdigital space with skin lesions that can result in deep necrosis and phlegmonous inflammations.

Infectious processes are possibly implicated in the pathogenesis of IH. Tissues affected with IH exhibit decreased microbial richness and diversity compared to healthy skin (Bay et al., 2018). An increased susceptibility for IH has been described depending on the number of parities and lactations. From first parity to later parities, IH frequency and estimates of heritability increase (van der Spek et al., 2015). In a longitudinal study in which cows that had been affected with IH were analyzed with respect to the point in time when they were first diagnosed with IH, it was found that about 50% of all cows that were susceptible were affected in their first lactation, another 25% in their second lactation and another 10%–15% in third lactation while the remaining cows only exhibited IH very late in life (Swalve et al., 2017).

Already in very early literature, it has been hypothesized that a genetic predisposition is the main cause for the development of IH (Götze, 1952). In more recent studies using data collected at time of hoof trimming, rather elevated estimates of heritabilities are found (Gernand et al., 2012; van der Spek et al., 2013; Pijl and Swalve, 2017) and these even amount to magnitudes of 0.30–0.40 when threshold models are used or estimates from a linear estimation are converted to the underlying scale (Swalve et al., 2011; Pijl et al., 2013; van der Spek et al., 2013; Perez-Cabal and Charfeddine, 2015). In a study estimating the odds ratio for cows to be affected by IH when comparing the status of female ancestors, it was found that the risk to be affected increases 8.5-fold when comparing cows with affected dams and grand-dams vs. cows with non-affected ancestors (Pijl and Swalve, 2017). Up to recently, evidence for a genetic predisposition on a molecular level has been scarce and inconclusive (Koenig et al., 2005; Gernand et al., 2012; van der Spek et al., 2013). In a very recent study, a number of chromosomal regions putatively involved in the etiology of IH have been identified (Croue et al., 2019).

Aim of the present study was to identify genes that might play a role in the development of IH by means of a genome-wide association study based on a case-control design. As a further step, genes in the identified regions were subject to sequencing for detection of single nucleotide polymorphisms. Finally, polymorphic sites were further analyzed with respect to differences in gene expression. A herd of dairy cows exhibiting an extreme prevalence of IH served as a basis for the study.

## Materials and Methods

### Ethical Statement

Clinical inspections and sampling of cattle were done during routine hoof trimming on farm with written owner consent. Samples were taken exclusively by local veterinarians. The collection of samples was approved by the Lower Saxony State Office for Consumer Protection and Food Safety (33.19-42502-05-17A196) according to §8a Abs. 1 Nr. 2 of the German Animal Protection Law.

### Clinical Investigations and Sample Collection

Clinical investigations were completely connected to the hoof trimming routine as practiced on the study farm. The hoof trimming routine consisted on visits of the hoof trimmer at intervals of approximately 3 months. At each visit, around half of the cows of the herd were subject to hoof trimming, i.e., two consecutive visits were supposed to cover the entire herd. The herd therefore was inspected at two consecutive hoof trimming events in October, 2016, and January, 2017. Another hoof trimming event in January 2018 was used to take 2mm fine needle biopsies (FNB) (Tru-Punch Sterile Disposable Biopsy Punch, Sklar Instruments, VWR, Germany) from hyperplastic interdigital skin of eight affected cows for RNA and protein analysis. Healthy control samples were collected from an abbatoir.

A total of 110 cattle was assessed at visits 1 (V1) and 2 (V2) (**Table 1**). Excluding animals that had been presented twice, i.e., at V1 and V2, n = 91 animals remained. During the visits, individuals were inspected visually, phenotypes were recorded and pictures were taken for documentation. Blood samples were taken for DNA extraction during V1 and V2 (n = 91).

**Table 1:** Frequency of interdigital hyperplasia (IH) affected cows during two farm visits.

Clin. inspection^a)^	Cattle (n = )	Interdigital hyperplasia affected (n = )	
		Type A (%)^b)^	Type B (%)^c)^
V1	58	34 (59)	22 (38)
V2	52	37 (71)	26 (50)
Total	110	71 (65)	48 (44)

a) V1, First farm visit in October 2016; V2, Second farm visit in January 2017. b) IH type A, At least one affected hind leg. c) IH type B, Both hind legs affected.

### Genome-Wide Association Study (GWAS) and Statistical Analysis

Genotyping of the 91 samples was performed using the Illumina BovineSNP50 BeadChip. Raw data were processed using GenomeStudio V2011.1 (Illumina, San Diego, USA). Final reports were imported into SVS 8.8.3 (Golden Helix, Bozeman, USA) and low quality SNPs were filtered if call rates < 90%, MAF <0.01 and Fisher´s HWE p < 0.0001 (based on controls). LD pruning was performed using default parameters. Samples were filtered with call rates < 0.95. Mitochondrial DNA and sex chromosomes were excluded from the analysis. After filtering 70 samples and 45,232 SNPs remained in the analysis. Genome-wide associations were calculated under an additive and dominant model (Duenk et al., 2017). Associations were regarded as genome-wide statistically significant above a threshold of -log_10_P = 5.47 (*p* = 0.05) (Benjamini and Yekutieli, 2001). Associations of markers (-log_10_P-value, y-axis) were plotted against their chromosomal positions (UMD3.1.1, x-axis).

All n = 91 cows from V1 and V2 were genotyped for rs43572154 and rs377953295. Genotype counts, genotype, and allele frequencies were calculated for both variants. Hardy-Weinberg test was calculated according to Rodriguez et al. (Rodriguez et al., 2009). Frequency distribution for alleles and genotypes vs. types of definitions of disease status (Type A IH, Type B IH) was calculated using both Chi-squared test and Fisher’ s Exact Test.

As the proportion of the genetic variance ideally should be estimated from a large sample to be drawn from the entire population, an approximation was used for the evaluation of the relative importance of each SNP in terms of the variance accounted for. A completely random threshold model was applied using a logit-link function with SNP taken as random and applying PROC Glimmix in SAS. With the residual variance under this model commonly taken as a fixed value of pi²/3 equal to 3.29, the proportion of the variance accounted for by each single SNP was estimated as [SNP variance/(SNP variance + residual variance)] * 100.

Based on the number of cattle analyzed here, a true population wide LD estimation between pairwise SNP cannot be performed. As an approximation, based on the genotypes for putatively functional SNP as identified after sequencing and the respective genotypes for SNP included in the array, a pairwise analysis was done by i) code the genotypes as 0, 1, 2 for both SNP in question and ii) estimate the correlation between the resulting variables and square it.

### Sanger Sequencing and Genotyping

DNA was extracted from blood samples using MagNA Pure LC DNA Isolation Kit I (Roche Diagnostics, Mannheim, Germany) or a modified salting out procedure (Miller et al., 1988). For Sanger sequencing of *ROR2*, primers were designed to amplify exons and intron–exon boundaries (**Supplementary Table S1**) (Sanger et al., 1977). PCR products were purified with Rapid PCR Cleanup Enzyme Set (New England Biolabs GmbH, Frankfurt am Main, Germany) and sequenced using the BigDye Terminator v3.1 Cycle Sequencing Kit (Applied Biosystems, Fisher Scientific GmbH, Schwerte, Germany) on an ABI PRISM 3130xl Genetic Analyzer (Life Technologies, Foster City, United States) according to the manufacturers´ protocols. The same primers were used for sequencing PCR products. SeqMan Pro software [version 15.0.0 (160) Intel, DNASTAR] was used for sequence alignments. Genotyping of variants NC_037335.1:g.85,905,534T > A and NC_037335.1:g.86,140,379A > G (ARS-UCD1.2) was done by diagnostic sequencing. To identify IH associated variants, *ROR2* was comparatively sequenced using eight IH affected (IHA) and eight healthy control cattle. Allele and genotype frequencies of variant rs377953295 were determined in 3,093 random Holstein cattle samples.

### RNA and Protein Preparation

For RNA and protein analysis samples were collected during the visits at the farm. A total of eight samples (3 x A_A, 3 x A_T, 2 x T_T) were collected for RNA and seven samples (3 x A_A, 3 x A_T, 1 x T_T) for protein analysis corresponding to the genotypes at variant rs77953295. Harvested tissue was immediately immersed in RNAlater (Ambion, ThermoFisher Scientific, Dreieich, Germany) and stored at 4°C. Tissue samples were homogenized in the Qiazol Lysis Reagent (Qiagen, Hilden, Germany). According to the user guide of TRIzol Reagent, total RNA was extracted and eluted in 30 µl RNase-free water and the concentration and purity were measured on a NanoDrop (ThermoFisher Scientific, Dreieich, Germany). 0.2 µg of RNA was converted to cDNA using Maxima H Minus First Strand cDNA Synthesis Kit with dsDNase (ThermoFisher Scientific, Dreieich, Germany).

Protein was extracted and solubilized in an optimized EDTA lysis buffer (Kopec et al., 2017). Protein concentrations were measured using the Bradford assay (Bradford, 1976). RNA and protein samples were stored at –80° until further use.

### *ROR2* Isoform Detection and Semi-Quantitative Real Time RT-PCR

Potential *ROR2* isoforms were analyzed in tissue samples of the interdigital region, lung and spleen of healthy cattle using primer pairs ROR2_cDNA_1/2_fwd (5´-CAGCCCTGTTCCAACTCTGA-3´), ROR2_cDNA_1/2_rev (5´-CCGTATTCCGTCTTGCGGAT-3´), ROR2_cDNA_2_fwd (5´-GGCATGGAGTACCTGTCCAG-3´), and ROR2_cDNA_2_rev (5´-GGCCAGGTCTTTGTGGACCA-3´).

Real-time quantitative PCR was performed using cDNA synthesized from RNA isolated from FNB of IHA cattle and controls. Statistical testing for mRNA expression normalized to *GAPDH* (glyceraldehyde 3-phosphate dehydrogenase) and β*-actin* was determined by the 2^-ΔΔ^ *^CT^* method using Microsoft Excel for Mac 2011 (Livak and Schmittgen, 2001). *GAPDH* was amplified using primers GAPDH_cDNA_fwd (5´-CCACTCCCAACGTGTCTGTT-3´) and GAPDH_cDNA_rev (5´-GCTTCACCACCTTCTTGATCTCATC-3´) (Macabelli et al., 2014). β*-actin* was amplified using primers ACTB_cDNA_fwd (5´-GTCATCACCATCGGCAATGAG-3´) and ACTB_cDNA_rev (5´-AATGCCGCAGGATTCCATG-3´) (Huang et al., 2013). Selection of the suitable reference gene was done according to the MIQE guidelines (Bustin et al., 2009). When comparing relative *ROR2* expression in 10 different tissue samples of a healthy Holstein cow, *GAPDH* showed the best invariant expression levels and was used as normalizer. When comparing relative *ROR2* expression only in interdigital skin tissue β-actin showed the best invariant expression levels and was therefore used as normalizer.

### Western Blot Analysis

Protein samples (10 µl) were mixed with 4X Bolt LDS Sample Buffer (Novex, ThermoFisher Scientific, Dreieich, Germany) supplemented with 10% 2-mercaptoethanol, and incubated at 70°C for 10 mins. Electrophoresis was performed on 8% Bolt Bis-Tris Plus Gels (Novex, Thermo Fisher Scientific) in Bolt MES SDS Running Buffer (Novex, ThermoFisher Scientific, Dreieich, Germany) at 165 V for 57 mins. Proteins were transferred to nitrocellulose membrane (ThermoFisher Scientific, Dreieich, Germany) at 15 V for 1 h in transfer buffer. After blocking overnight at 4°C, immunoblots were incubated with primary anti-ROR2 antibody (1:500, ABIN2706970, Cohesion Biosciences, Aachen, Germany) and anti-β-actin (1:10,000, A5441, Sigma Aldrich, Darmstadt, Germany) at room temperature for 1 h. Incubation with the secondary antibodies (1:5000 for #1706515, 1:10,000 for #1706516, Bio-Rad, Munich, Germany) was done at room temperature for 1 h. Immunoblots were developed with Western ECL (GERPN2109, Sigma Aldrich, Darmstadt, Germany). Images from Western blots were captured and quantification was performed with ImageJ software (Schneider et al., 2012).

## Results

### Identification of an IH Associated Region on BTA8 Using a Genome-Wide Association Study

**Figure 1:**
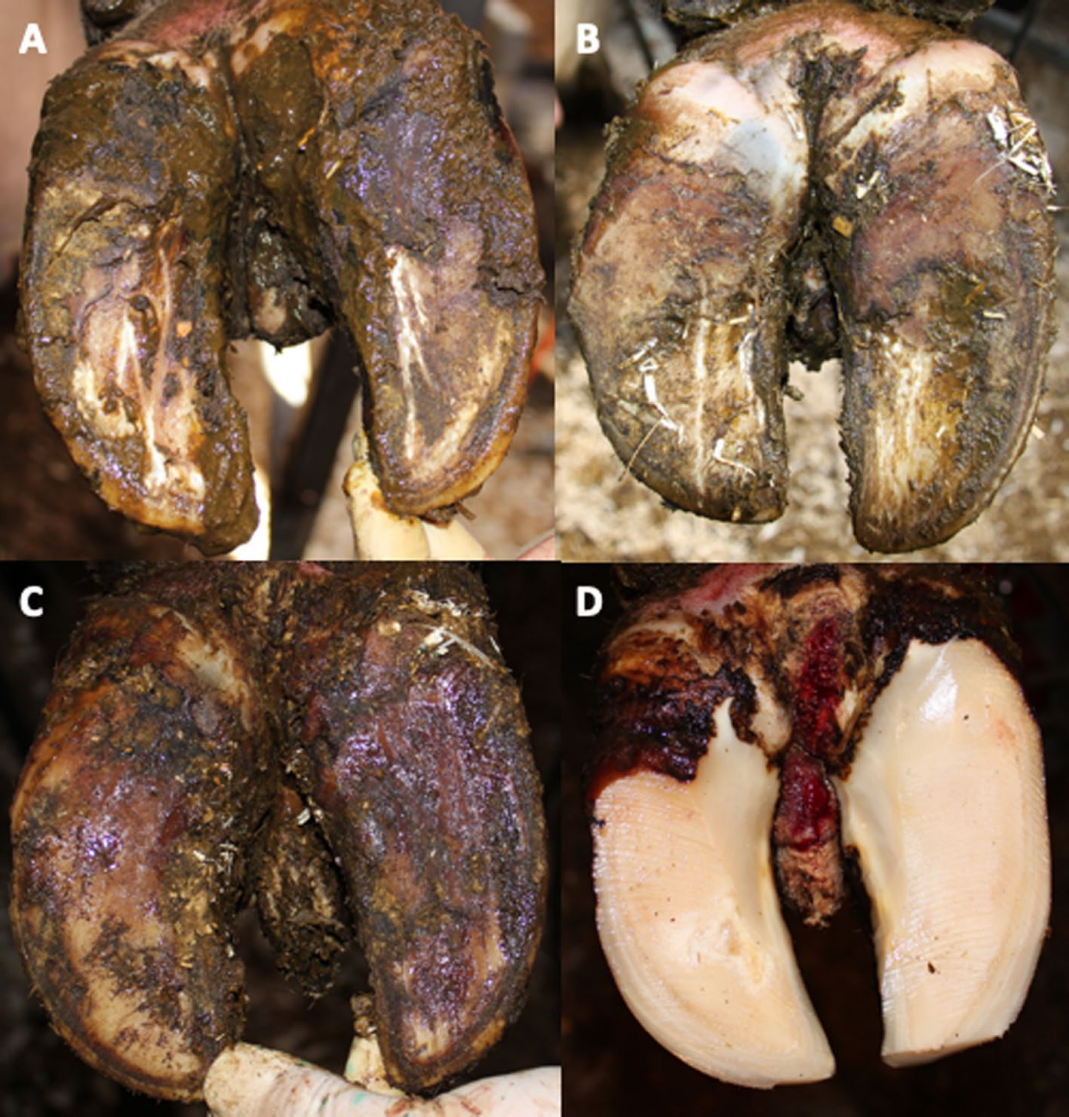
Clinical signs of bovine interdigital hyperplasia. **(A–D)** show different developmental stages with increasing size of dermal hyperplasia found in the interdigital space with skin final skin lesions **(D)**. **(D)** Interdigital hyperplasia after hoof trimming.

**Figure 2:**
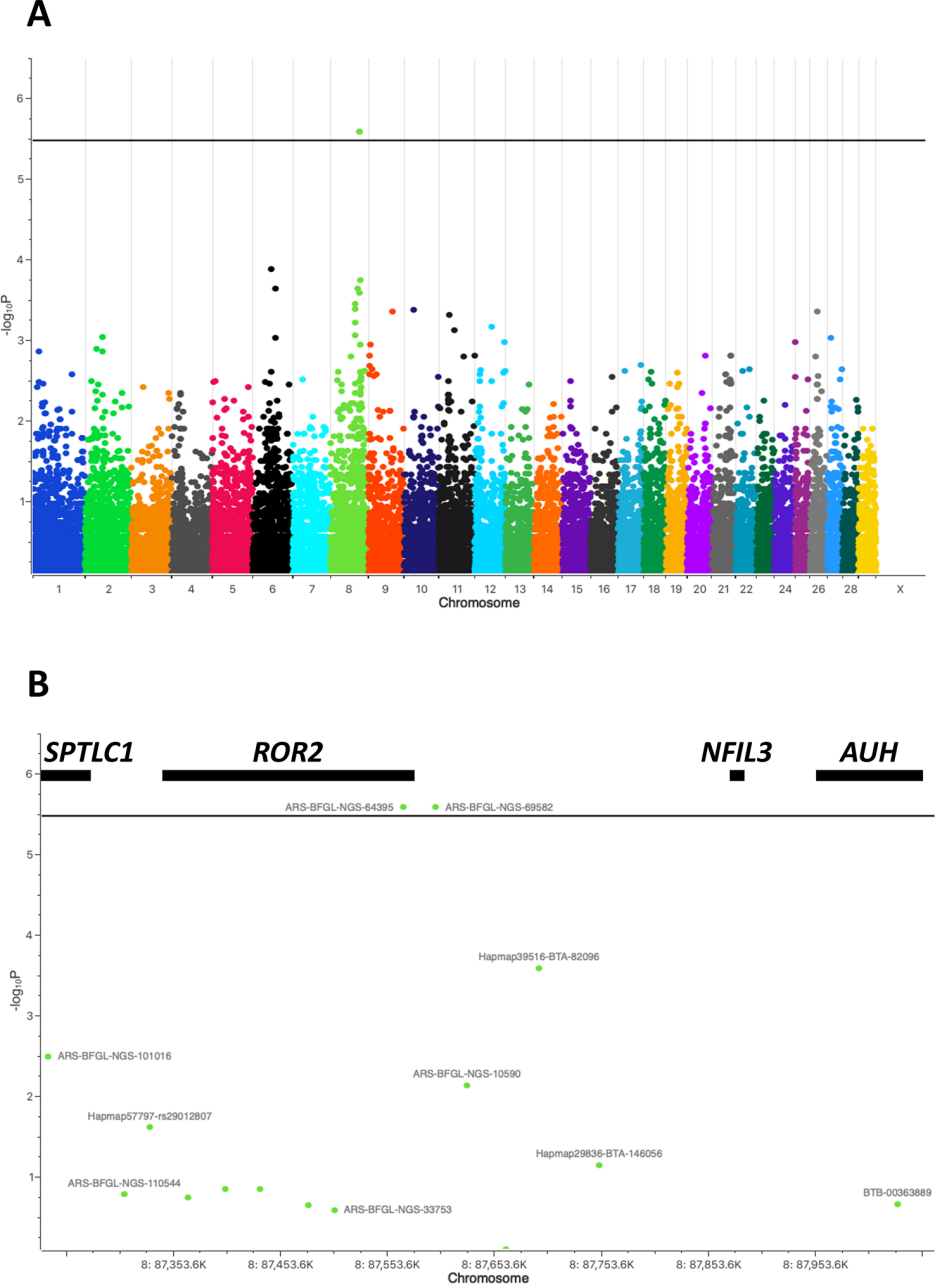
Manhattan plot of genome-wide association study for bovine interdigital hyperplasia. **(A)** Marker associations are plotted as negative log-transformed *P* values against the position in the bovine genome (UMD3.1.1). Two markers ARS-BFGL-NGS-64395 and ARS-BFGL-NGS-69582 exceed the genome-wide significance threshold of -log_10_P = 5.47 (black line). **(B)** Enlargement of the associated chromosomal region on BTA8 flanking markers ARS-BFGL-NGS-64395 and ARS-BFGL-NGS-69582. Genes located within this region, i.e., *SPTLC1, ROR2, NFIL3,* and *AUH*, are shown with black bars at their approximate positions.

During V1 and V2 a total of 110 cattle was clinically inspected (**Figure 1**) and blood samples drawn (**Table 1**). After removing duplicates an initial set of 91 samples remained. After quality filtering of genotyping data 70 cattle were used for the genome-wide association study. Depending on the type of IH 41 cases/29 controls (type A) or 29 cases/41 controls (type B) were analyzed using an additive and/or dominant genetic model. For both IH types and genetic models markers on BTA8, i.e., ARS-BFGL-NGS-64395, ARS-BFGL-NGS-69582, showed highest -log_10_P values above a chromosome-wide significance threshold of -log_10_P = 5.47 (*p* = 0.05) (**Figures 2A, B**). ARS-BFGL-NGS-64395 is located in intron 5 and ARS-BFGL-NGS-69582 downstream of the *ROR2* gene (tyrosine-protein kinase transmembrane receptor) (**Figure 2B**). DNA sequence comparison revealed two variants in the coding region of *ROR2* in exon 1 (NC_037335.1g.85905534T > A; rs377953295; ARS-UCD1.2) and exon 9 (NC_037335.1g.86140379A > G; rs43572154; ARS-UCD1.2). Estimates of r² for pairwise comparisons of rs377953295 with significant SNP included in the BeadChip were r² = 0.973 (for ARS-BFGL-NGS-64395), r² = 0.920 (for ARS-BFGL-NGS-69582) and r² = 0.652 (for Hapmap39516-BTA-82096). It is especially notable that, with the exception of one animal, rs377953295 and ARS-BFGL-NGS-64395 appeared to be in perfect linkage.

The variant in exon 1 resulted in an amino acid exchange from tryptophan to arginine (ENSBTAP00000053765.2:p.Trp9Arg) located in the N-terminal signal peptide. The variant in exon 9 caused an amino acid exchange from methionine to valine (ENSBTAP00000053765.2:p.Met901Val) at the C-terminal intracellular end outside of any functional domain (Rebagay et al., 2012).

### Genotyping and Association Analysis of *ROR2* Variants

To determine the genotype frequencies of rs377953295 (exon 1) and rs43572154 (exon 9) in IHA and control cattle (IHF) both variants were genotyped in all clinically inspected cattle. Results are summarized in **Table 2**. Although for both SNPs the analyzed cohorts were in Hardy-Weinberg equilibrium, it was striking that the A_A-genotype (rs377953295) was not detected in healthy individuals. Fisher´s exact test using a 2x3 contingency table showed that the genotype distribution for this SNP was significantly deviating from expectation (p = 4.9e-4), whereas the distribution of genotypes for rs43572154 (exon 9) did not deviate. SNP rs43572154 (exon 9) was therefore excluded as potential causative variant. Association of rs377953295 (exon 1) was further investigated comparing the frequency distribution of the three genotypes with the different clinical IH types in a total of 94 cattle. The allele and genotype frequency of rs377953295 was also determined in 3,093 random Holstein cattle samples. The genotype count in this cohort was 2,276 (T_T), 622 (A_T), and 195 (A_A) resulting in allele frequencies of 0.84 (T-allele) and 0.16 (A-allele). The proportion of the total variance accounted for by SNP rs377953295 was estimated to be 32.2% for type A IH and 20.1% for type B IH.

**Table 2:** Genotype frequencies of the receptor tyrosine kinase-like orphan receptor 2 (*ROR2*) variants rs377953295 (exon 1) and rs43572154 (exon 9) in interdigital hyperplasia (IH) type A^a)^ affected and free (= healthy) cattle.

	rs377953295 (exon 1)			rs43572154 (exon 9)		
	T_T	A_T	A_A	A_A	A_G	G_G
IHF^b)^	32	5	0	1	10	15
IHA^c)^	23	23	7	3	17	34
Total	55	28	7	4	27	49
HWE^d)^ (χ ^2^)	1.53			0.01		

a) In type A IH, At least one affected hind leg.

b) IHF, Interdigital hyperplasia free.

c) IHA, Interdigital hyperplasia affected.

d) HWE: Hardy-Weinberg equilibrium of total cohort.

As shown in **Table 3** the presence of the A_A-genotype significantly associated with the different clinical forms of IH. Differences between type A and B IH are detectable in the distribution of genotypes T_T and A_T. Furthermore, it is evident that heterozygous individuals are more often affected which suggests that the A-allele at rs377953295 (exon 1) seems to be associated with an increased risk to develop IH.

**Table 3:** Statistical evaluation of SNP rs377953295 (exon 1) as causative variant for type A and type B interdigital hyperplasia (IH).

	Type A IH^a)^			Type B IH^b)^		
	T_T	A_T	A_A	T_T	A_T	A_A
IHF^c)^	21	4	0	35	6	0
IHA^d)^	38	24	7	24	22	7
Total	59	28	7	59	28	7
F-statistic	7.16			16.94		
P-values (χ^2^, FET^e)^)	0.0279, 0.0026			0.0002, < 0.0001		

a) In type A IH: IHF, no IH; IHA, at least one IH at one hind leg.

b) In type B IH: IHF, no IH or only one IH; IHA, IH at both hind legs.

c) IHF: Interdigital hyperplasia free.

d) IHA: Interdigital hyperplasia affected.

e) FET: Fisher´s exact test.

In summary, the genome-wide association study identified a significantly associated chromosomal region on BTA8 harboring a potential candidate gene with a missense variant that is significantly overrepresented in IHA cattle. As the variant rs377953295 (exon 1) results in an amino acid exchange at position nine of the ROR2 signal peptide it was hypothesized that this could influence expression and finally an insufficient translocation of ROR2 into the plasma membrane.

### Expression of *ROR2* in the Interdigital Skin Is Significantly Downregulated in Affected Homozygous Animals Harboring the A-allele at rs377953295

**Figure 3:**
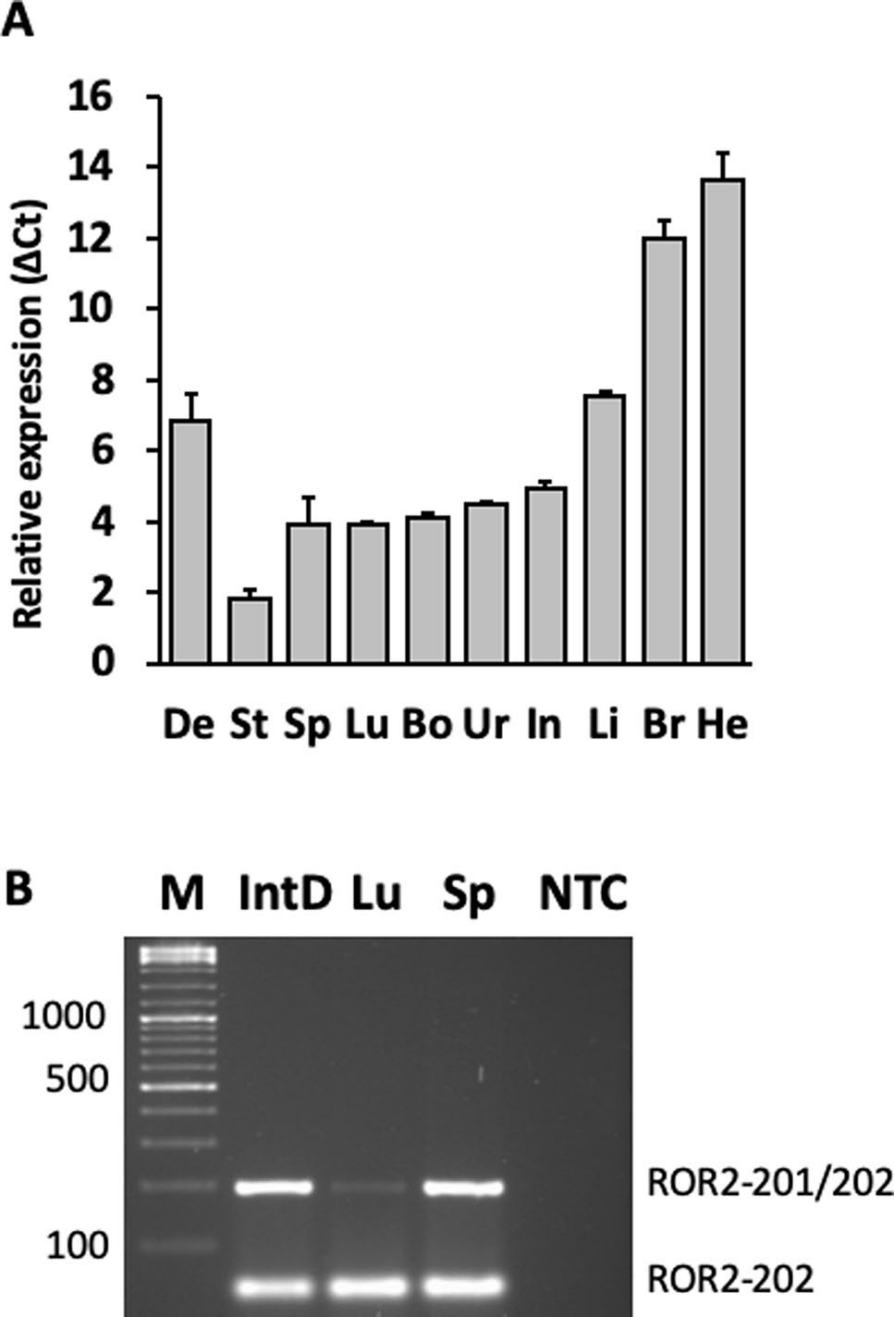
Detection of the receptor tyrosine kinase-like orphan receptor 2 (*ROR2*) transcripts and isoforms in different bovine organs and tissues. **(A)** Organ and/or tissue samples (De, interdigital dermis, He, heart, Br, brain, Li, liver, Int, intestine, Ur, urinary bladder, Bo, bone marrow, Lu, lung, Sp, spleen, St, stomach) were collected from healthy cattle and RNA extracted. Relative expression levels were calculated using the average Δ Ct-values of three biological replicates with GAPDH as normalizer. **(B)** *ROR2* isoform specific primers were used to amplify RNA from IntD, interdigital dermis, Lu, lung and Sp, spleen (NTC, non template control). Two amplicons corresponding to isoform ROR2-202 and isoforms ROR2-201/202 together can be seen. Amplicons were separated on a 2% agarose gel and visualized using Ethidium bromide staining.

**Figure 4:**
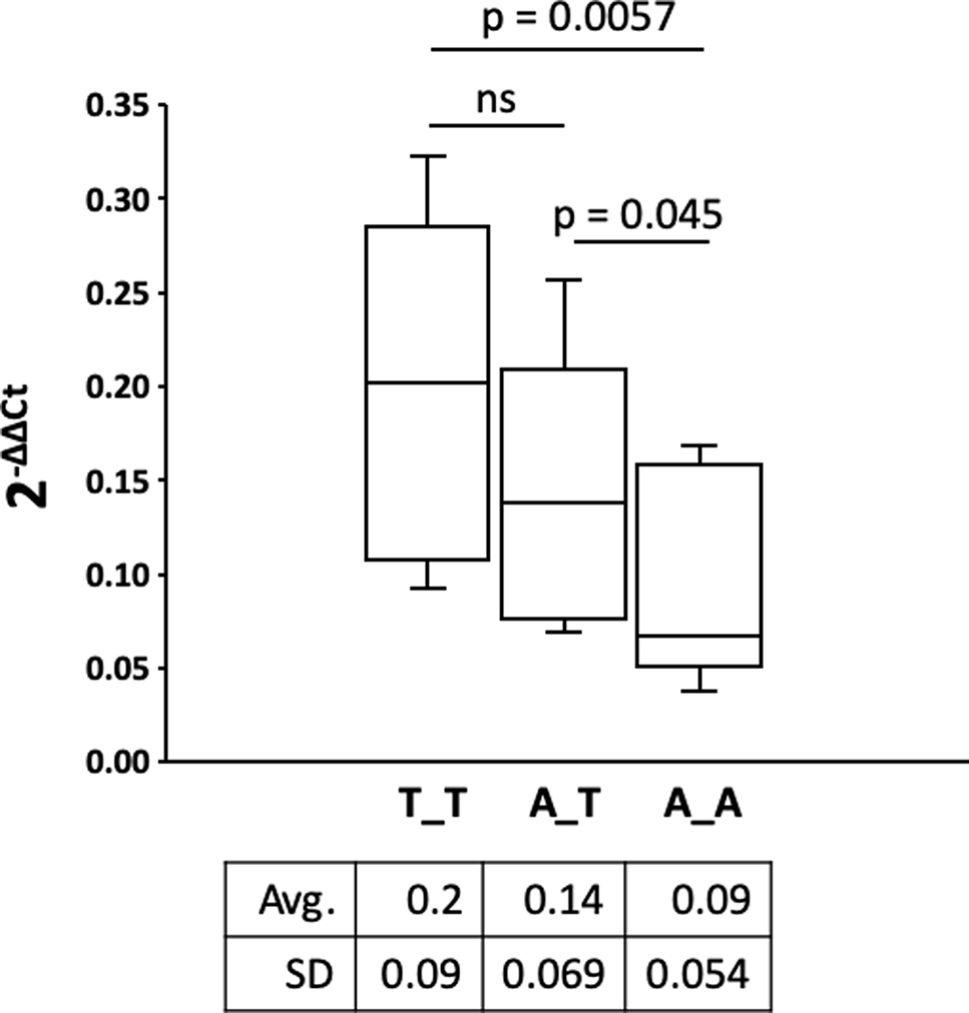
Comparison of the receptor tyrosine kinase-like orphan receptor 2 (*ROR2*) expression in hyperplastic interdigital skin tissue. Interdigital skin tissues were collected from 3 A_A, 3 A_T, 2 T_T cattle using fine needle biopsies (FNB). Genotypes A_A, A_T and T_T correspond to the missense variant in exon 1 (rs377953295). Two samples were taken from healthy control cattle as reference. Expression of *ROR2* and internal control was done by real-time quantitative PCR. Calculation of *ROR2* expression fold change in the IH affected cattle was done using the 2^-ΔΔCt^ method with β-actin as normalizer and the average expression of *ROR2* in the healthy controls as reference (Livak and Schmittgen, 2001). *ROR2* expression fold change of the three genotypes is shown as box-and-whisker plot (whiskers indicate minima and maxima). The vertical line depicts the median. Significance was calculated using a one-tailed t-test with p < 0.05.

**Figure 5:**
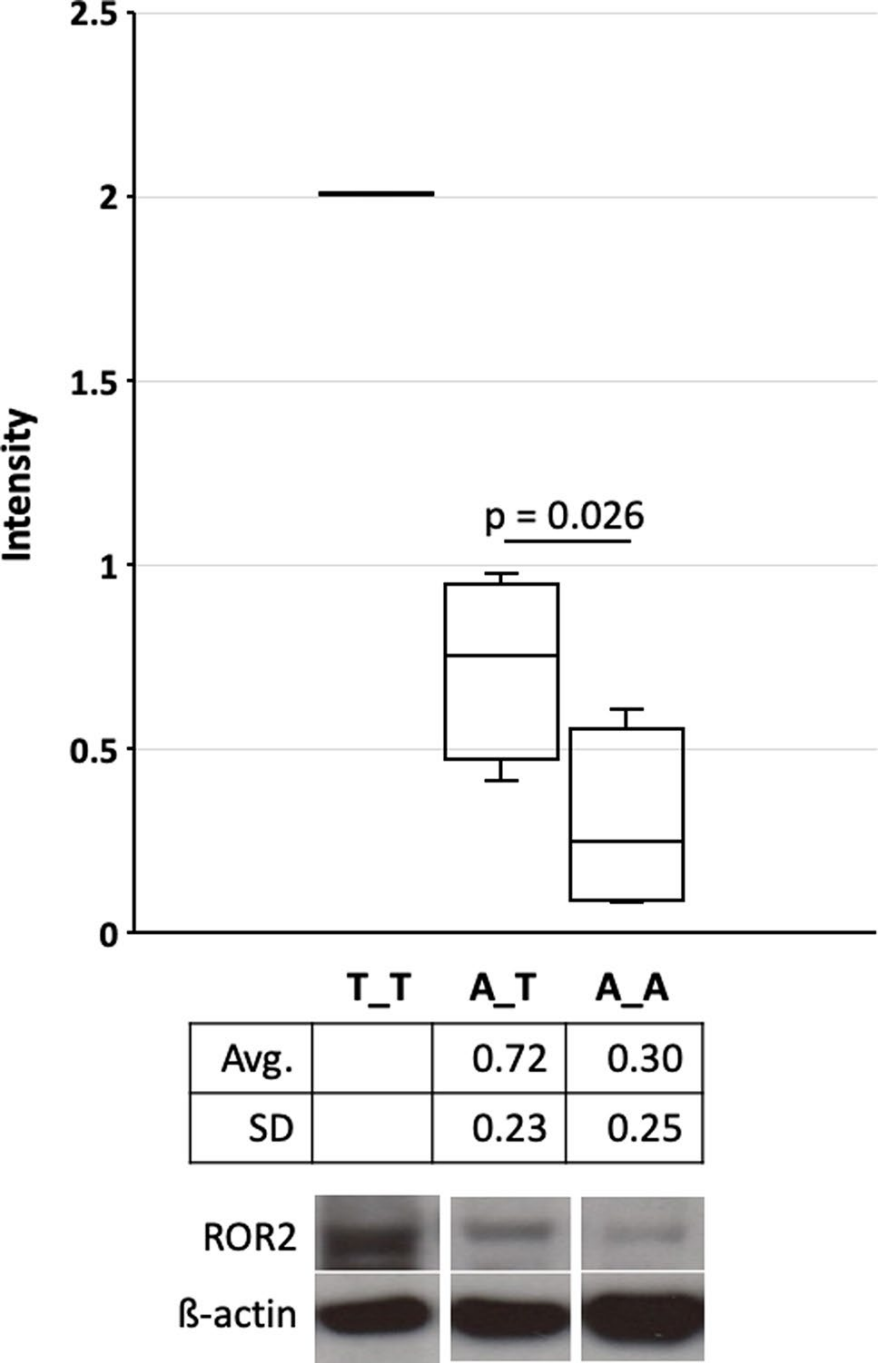
Quantification of the receptor tyrosine kinase-like orphan receptor 2 (ROR2) protein in hyperplastic interdigital skin tissue. Interdigital skin tissues were collected from 3 A_A, 3 A_T, 1 T_T cattle using fine needle biopsies (FNB) (see also **Figure 4**). Genotypes A_A, A_T and T_T correspond to the missense variant in exon 1 (rs377953295). Two samples were taken from healthy control cattle as reference. Protein extracts were separated on 8% Bolt Bis-Tris Plus gels and transferred to nitrocellulose membranes. Membranes were incubated with primary anti-ROR2 antibody and anti-β-actin. Blots were developed with Western ECL. Images were captured and intensity quantification was performed with ImageJ software (Schneider et al., 2012). Significance was calculated using a one-tailed t-test with p < 0.05. As only one samples for genotype T_T were available statistical significance, average and standard deviation is not depicted. Whiskers indicate minima and maxima. The vertical line depicts the median.

*ROR2* is a classical housekeeping gene harboring a GC-rich and TATA-less promoter (Yella and Bansal, 2017). Therefore, it was expected that *ROR2* is widely expressed as shown in humans (Thul and Lindskog, 2018). However, as *ROR2* has also been shown to be mainly expressed in early and expression abates during later embryonal development, it was first of all important to demonstrate *ROR2* expression in tissues of adult cattle (Matsuda et al., 2001; Yoda et al., 2003). Therefore, 10 different tissues/organs, i.e., interdigital skin, lung, spleen, brain, heart, liver, stomach, intestine, urinary bladder, and bone marrow, were collected at an abattoir and analyzed. As shown in **Figure 3A** expression was detected at different levels in all analyzed samples. Importantly, *ROR2* was expressed in the skin of the interdigital region further supporting the genomic data that it could be involved in the development of IH. In addition, it has been reported that two isoforms of *ROR2* (ROR2-201, ROR2-202) exist differing in the usage of two alternative first exons. Only ROR2-201 harbors the exon with variant rs377953295 and therefore it was necessary to prove that this isoform was expressed in the interdigital skin. Using an isoform specific PCR it was possible to show that both isoforms are expressed in the interdigital skin (**Figure 3B**). Finally, the association of the three genotypes of variant rs377953295 with the expression of *ROR2* in the interdigital skin was tested. For this purpose samples of the hyperplastic interdigital skin of cattle harboring the T_T-, A_T- and A_A-genotypes at rs377953295 were compared using interdigital skin of healthy cattle as normalizer. As depicted in **Figure 4** the different genotypes within the IHA samples showed significant reductions between genotype T_T vs A_A as well as A_T vs A_A. The difference between T_T vs A_T was not significant. The differences in *ROR2* mRNA levels were also reflected in the ROR2 protein levels (**Figure 5**). Note, that for the protein extraction of the T_T genotype only one hyperplastic skin sample was available and therefore a statistical verification was omitted. For reasons of animal welfare we refrained from additional sample collections. However, the ROR2 protein amount in the T_T sample was clearly above the other two genotypes.

In summary, the functional analyses are in agreement with the genomic data that a reduction of *ROR2* expression on RNA and protein level seems to be associated with the risk to develop IH and is highest in cattle with the A_A genotype at rs377953295.

## Discussion

IH is a serious health issue in cattle production. Besides the clinical and animal welfare relevance it also has tremendous effects on the general performance of the animal. Therefore, it is of great importance to elucidate the molecular genetics of IH and find potential causative variants that could be used for selection in future breeding programs.

In the study described here a herd with a high prevalence of IH was identified which was followed over a longer period of time allowing a repeated thorough clinical inspection and sampling during professional hoof trimming. A GWAS was performed showing two genome-wide significantly associated SNP loci at around 87.5Mb on BTA8. In a previous study, 17 suggestive associations (p < 0.20) spreading across the bovine genome had been detected for IH in Holstein cattle (van der Spek et al., 2015). Although five of them were also located on BTA8 (8Mb, 24Mb, 25MB, 43Mb), the IH associated region in this study was located further downstream. Fortunately, only four genes were located in the direct chromosomal proximity of the associated SNPs (**Figure 2B**). Genes flanking *ROR2*, i.e., *SPTLC1* (long-chain base subunit 1 of serine palmitoyltransferase) (Suh et al., 2014; Ho and Jerath, 2018), *NFIL3* (interleukin 3-regulated nuclear factor) (Kobayashi et al., 2011; Kobayashi et al., 2014), and *AUH* (3-methylglutaconyl-CoA hydratase) (Ijlst et al., 2002; Ly et al., 2003) were excluded as potential candidates as they have been reported in humans or mice to be causative for hereditary sensory and autonomic neuropathy (type 1A), susceptibility to inflammatory bowel disease and 3-methylglutaconic aciduria (type 1), respectively. On the other hand, as *ROR2* had been associated with terminal limb malformations in humans including cutaneous syndactyly, it seemed to be a reasonable candidate for further analysis (Oldridge et al., 2000; Schwabe et al., 2000; Afzal et al., 2000; van Bokhoven et al., 2000; Bacchelli et al., 2003; Tufan et al., 2005; Ali et al., 2007; Brunetti-Pierri et al., 2008). Additionally, one of the associated SNPs was directly located within the gene (intron 5). A DNA sequence comparison of *ROR2* in IHA and healthy cattle revealed two missense variants in exon 1 and exon 9, respectively. However, only the variant in exon 1 (rs377953295) resulting in an amino acid exchange in the signal peptide of *ROR2* (ENSBTAP00000053765.2:p.Trp9Arg) remained significantly associated with IH after screening the cohort of cattle at the farm. So far *ROR2* variants have not been associated with any other disorder or trait in cattle and therefore the identification of the missense variant was a novel finding.

But also regarding its biological function, *ROR2* was obviously an interesting candidate. ROR2 belongs to the receptor tyrosine kinases (RTK), a large superfamily of transmembrane glycoproteins. Previous studies have shown that ROR2 is important for the formation of the distal limbs (Takeuchi et al., 2000; Matsuda et al., 2001). Molecular genetic analyses in humans have revealed that mutations in *ROR2* cause dominant Brachydactyly type B and recessive Robinow Syndrome, with terminal limb malformations as common symptoms (Oldridge et al., 2000; Schwabe et al., 2000; Afzal et al., 2000; van Bokhoven et al., 2000; Bacchelli et al., 2003; Tufan et al., 2005; Ali et al., 2007; Brunetti-Pierri et al., 2008; Kjaer et al., 2009). Defects of the distal limbs have also been observed in *ROR2* ^-/-^ mice, mainly due to abnormal cartilage and growth plate development, as well as ossification (Takeuchi et al., 2000; DeChiara et al., 2000). Hence, the reported functional role, determined positional association and identification of a missense variant in *ROR2* in IHA cattle were convincing enough to extend the analyses.

Therefore, *ROR2* expression was analyzed in the interdigital skin. Although earlier studies indicated that *ROR*2 expression in adults was restricted to parathyroid, testis, and uterus, it is now known that its expression may be more widespread than originally thought (Katoh and Katoh, 2005; Thul and Lindskog, 2018). During embryogenesis *ROR2* expression was identified in heart, lungs, face, and limbs (Matsuda et al., 2001). Our data show that different amounts of *ROR2* mRNA can be detected in interdigital skin, lung, spleen, brain, heart, liver, stomach, intestine, urinary bladder, and bone marrow of adult cattle. In addition, two *ROR2* isoforms (ROR2-201, ROR2-202) were detected differing in the usage of an alternative first exon. Especially isoform ROR2-201 harboring the exon with the missense variant was detectable in the interdigital skin. Although compared to other organs and/or tissues expression of *ROR2* in the interdigital skin was intermediate, the identification of *ROR2* transcripts was important to support its assumed role in IH development. Furthermore, the relative amount of *ROR2* transcripts significantly differed between the three genotypes in correlation with the IH status. Homozygous A_A IHA cattle showed significantly reduced *ROR2* expression in the hyperplastic interdigital skin, which is also reflected in the amount of ROR2 protein. In addition, *ROR2* expression in hyperplastic compared to normal interdigital skin showed a significant reduction, which explains the observation that hyperplastic skin alterations were also present in T_T or A_T cattle. The down-regulation of *ROR2* could be explained by a general mechanism of transcriptional quality control. For instance, when mutant signal sequences fail to bind to the signal recognition particle at the ribosome exit site, the nascent chain instead contacts Argonaute2 and the mutant mRNAs are specifically degraded (Karamyshev et al., 2014). Specific mRNA degradation preemptively regulates aberrant protein production (Karamyshev et al., 2014). However, other mechanisms resulting in the general down-regulation of *ROR2* in hyperplastic interdigital skin have to be taken into account (Roman-Gomez et al., 2007; Lara et al., 2010).

In the etiology of IH, the role of ROR2 in signal transduction seems to be important. The main histological alterations in hyperplastic interdigital skin are proliferating fibroblasts, multiplex papilliferous epidermal ridges as well as increased cellularity in the *stratum granulosum* and *stratum spinosum* (Amstel and Shearer, 2006; Kashyap et al., 2017). In this respect, it is important to note that Wnt signaling pathways play crucial roles in the regulation of skin development and epidermal stem cells behavior (Clevers et al., 2014; Veltri et al., 2018). Sustained epidermal activation of Wnt/β-catenin signaling not only stimulates fibroblast proliferation, but also causes structural remodeling of the entire dermis (Collins et al., 2011). Binding of Wnt isoforms to Fz-LRP complex (frizzled-low density lipoprotein receptor-related protein) generates β-catenin signaling, whereas binding to the atypical receptor tyrosine kinase ROR2 can inhibit this activity (Gordon and Nusse, 2006). The significant down-regulation of ROR2 could affect the inhibition of the canonical Wnt pathway, resulting in the abnormal cellular processes related to aberrant epidermal development. This potential biochemical effect is consistent with our finding that ROR2 seems to be an IH suppressor and that decreased expression level of *ROR2* leads to IH development. Noteworthy, disease suppressive relationships of ROR2 have been described in hepatocellular cancer, colon cancer, and hematological malignancies (Roman-Gomez et al., 2007; Lara et al., 2010; Yuan et al., 2011; Geng et al., 2012). The canonical Wnt pathway has a pro-tumorigenic effect, leading to a series of cellular processes including proliferation, differentiation, polarity, migration, invasion, adhesion, and survival (Chien et al., 2009; Ford et al., 2013). Through inhibiting this canonical Wnt signaling as a gatekeeper, ROR2 has been proposed to play a role in tumor suppression (Ford et al., 2013). However, further studies will be required to clarify the exact molecular mechanism caused by down-regulation of *ROR2* expression in IH development.

## Data Availability Statement

Publicly available datasets were analyzed in this study. This data can be found here: https://www.ncbi.nlm.nih.gov/genome/gdv/browser/?context=genome&amp;acc=GCF_002263795.1, https://www.ensembl.org/Bos_taurus/Gene/Summary?g=ENSBTAG00000005092;r=8:85905346-86141520;t=ENSBTAT00000061589, https://www.proteinatlas.org, http://www.ncbi.nlm.nih.gov/sites/entrez?db=OMIM, http://www.informatics.jax.org/marker/MGI:1347521.

## Ethics Statement

The animal study was reviewed and approved by Lower Saxony State Office for Consumer Protection and Food Safety (33.19-42502-05-17A196). Written informed consent was obtained from the owners for the participation of their animals in this study.

## Author Contributions

HS and BB conceived and designed the overall study. XZ carried out the molecular experiments. HS, FR, and RP performed the clinical inspections and sample collection at the farm. XZ, HS, MW-D, and BB carried out the bioinformatics analysis. XZ and BB wrote the initial draft and subsequently all authors contributed to the presentation of the results. All authors read and confirmed the final version of the manuscript.

## Conflict of Interest

The authors declare that the research was conducted in the absence of any commercial or financial relationships that could be construed as a potential conflict of interest.
