# Supplementary material for: Interdigital Hyperplasia in Holstein Cattle Is Associated With a Missense Mutation in the Signal Peptide Region of the Tyrosine-Protein Kinase Transmembrane Receptor Gene
Source: Front Genet. 2019 Nov 13;10:1157. doi: 10.3389/fgene.2019.01157 (PMC6863962; doi:10.3389/fgene.2019.01157)
Supplement: Supplementary file 1 [file Table_1.docx]

**Table S1** *ROR2* primers for PCR and Sanger sequencing

| **Primer name** | **5´- 3´** | **Product size (bp)** |
| --- | --- | --- |
| ROR2_Ex1fwd | CGAAAGGGACCAACTTGCCA | 282 |
| ROR2_Ex1rev | CATCGCGAGGGAAGGGACG |  |
| ROR2_Ex2fwd | GGGCAGTTTTGCTAAAACACTAT | 300 |
| ROR2_Ex2rev | GGGAGACCCTGACCATCCAT |  |
| ROR2_Ex3fwd | GTGGTGGAGGCAACATTCTA | 617 |
| ROR2_Ex3rev | TCATGCACAATGGGAAAGGC |  |
| ROR2_Ex4fwd | CAGAGAGCACCCCTTCCATC | 370 |
| ROR2_Ex4rev | TGCCATCCCTGTGTGAAGTC |  |
| ROR2_Ex5fwd | GCCAAGGGACAAGATGGCTA | 330 |
| ROR2_Ex5rev | GGGACAAAATACACAAATGAGACTG |  |
| ROR2_Ex6fwd | CAGTTGCAAATCTGGGCGG | 668 |
| ROR2_Ex6rev | GAATGGAGCGGGTCTGTG |  |
| ROR2_Ex7fwd | TGGCGAGGTGGTTTGGTTAT | 479 |
| ROR2_Ex7rev | ATGGTGGTGAAACACGGTGG |  |
| ROR2_Ex8fwd | AGTTGGAGGTGGGAGTGGGC | 346 |
| ROR2_Ex8rev | GTAGAGGTTAAGCCTGGGGG |  |
| ROR2_Ex9.1fwd | CCGCCCAACCCCTTCTCC | 552 |
| ROR2_Ex9.1rev | GGCCAGGTCTTTGTGGACCA |  |
| ROR2_Ex9.2fwd | GGCATGGAGTACCTGTCCAG | 965 |
| ROR2_Ex9.2rev | GTCTCAGGGACTGAGCCG |  |
| ROR2_Ex9.3fwd | CTCCCATCACAGCGGCAGCG | 399 |
| ROR2_Ex9.3rev | AGGTGGGCACAGCGCAGCTC |  |
